# A systematic review of methodological approaches and measurement of adherence to micronutrient supplementation among women of reproductive age in low- and middle-income countries

**DOI:** 10.1186/s12889-025-24944-x

**Published:** 2025-12-13

**Authors:** Bee-Ah Kang, Jordyn Britton, Olajumoke Kiito Olarewaju, Aditi Luitel, Daryl Stephens, Rajiv N. Rimal

**Affiliations:** 1 Department of Health, Behavior and Society, Johns Hopkins Bloomberg School of Public Health, 615 N. Wolfe St., Baltimore, MD 21215 USA; 2 Department of Population, Family and Reproductive Health, Johns Hopkins Bloomberg School of Public Health, 615 N. Wolfe St, Baltimore, MD 21215 USA; 3 Howard University College of Medicine, 2400 6th St NW, Washington, DC, 20059 USA; 4 Department of International Health, Johns Hopkins Bloomberg School of Public Health, 615 N. Wolfe St, Baltimore, MD 21215 USA; 5 Albert Einstein College of Medicine, 1300 Morris Park Ave, Bronx, NY 10461 USA

**Keywords:** Adherence, Compliance, Maternal nutrition, Micronutrient supplementation, Micronutrient, Women of reproductive age, Nutritional behavior

## Abstract

**Background:**

Ensuring adherence to recommended micronutrient supplementation regimens is essential for addressing malnutrition among women in low- and middle-income countries (LMICs). While efficacy trials often use biomarker changes as proxies for adherence, public health programs employ diverse methodologies to measure adherence. However, consolidated evidence on the methodological approaches used in these programs and the extent of their standardization remains scarce. This systematic review addresses this gap by exploring adherence measurement methodologies, including adherence thresholds, data collection methods, the number of methods utilized, and measurement frequencies.

**Methods:**

We conducted a literature search on PubMed, Scopus, and Embase. Studies that measured adherence to WHO-recommended micronutrient supplements among women aged 15–49 in LMICs were included. Two reviewers screened titles/abstracts and full texts independently. Fifty-nine articles met the criteria. Data extraction involved four researchers. Article quality was assessed using Cochrane tools.

**Results:**

We categorized the included studies into behavioral vs. non-behavioral interventions to identify unique patterns between the two. Across non-behavioral studies, adherence was frequently defined with a threshold of 80%. These studies commonly used pill count to measure adherence and conducted frequent monitoring. Behavioral studies often adopted more diverse thresholds (e.g., 70%, 90 tablets), while 67% of them did not provide a clear threshold to define adherence. Self-report was the most commonly used measurement approach. The use of data triangulation and regular monitoring bolstered the validity of self-reports.

**Conclusions:**

Micronutrient supplementation programs would benefit from adopting evidence-based thresholds to define adherence levels, enhancing comparability across studies. Using a combination of methods and cross-checking data from multiple sources can improve the validity of adherence measurements. We recommend against treating adherence as a static, standalone construct; instead, reporting its trajectory throughout an intervention and its association with health outcomes can provide valuable insights for program planning. In terms of study characteristics, most studies have primarily focused on pregnant and lactating women, leaving adherence measurement approaches among nonpregnant women and adolescents largely understudied. We strongly encourage future research to investigate how methodological issues of adherence is uniquely experienced among this underserved population.

**Supplementary Information:**

The online version contains supplementary material available at 10.1186/s12889-025-24944-x.

## Background

Low- and middle-income countries(LMICs) disproportionately bear the burden of nutritional deficiencies among women. On average, 63% of women of reproductive age living in LMICs are vitamin D deficient, 41% are zinc deficient, and 23% are folate deficient, in addition to presenting inadequate levels of iron, B12, iodine and calcium [1]. Micronutrient deficiencies among women, especially during pregnancy, can lead to immediate and long-term adverse health outcomes, such as maternal mortality, pregnancy loss, low birth weight, as well as an increased risk of cardiovascular diseases and cognitive impairments later in life [1, 2]. According to the WHO’s dietary and micronutrient supplementation recommendations for pregnant women in antenatal care (ANC) [3] and for menstruating adult women and adolescent girls [4], providing micronutrient supplements has been a practical and cost-effective strategy for achieving better pregnancy, maternal, and child health outcomes globally.

Ensuring consistent uptake of recommended micronutrient supplements among individuals (such as Vitamin A, folate, and calcium for pregnant women), therefore, has been as a critical prerequisite for achieving optimal health and programmatic goals, especially in LMICs where dietary inadequacies are widespread. Adherence has been defined as “the extent to which a patient’s behavior matches the agreed recommendations from a healthcare provider” [5]. Although there is a tendency to dichotomize participants as being “adherent” or “non-adherent” in health research, no scientific consensus currently exists on what constitutes satisfactory adherence, as some studies define adherence as high as 90% of the prescribed supplements [6], while others use a lower threshold, such as 65% [7]. Also, heterogeneity in measurement methods and frequency may affect programs’ ability in defining adherence and interpreting intervention outcomes.

Given the intake requirements that differ by the type of micronutrient and the population, along with contextual factors that affect women’s adherence to supplementation in LMICs, it is critical for micronutrient supplementation programs to properly define, measure, and assess adherence, ultimately to accurately examine the impact of the interventions on health outcomes. This is necessary not only to advance the field of adherence research in nutrition, but also to guide program implementers and funders to make informed decisions on implementing and investing in supplementation programs. Prior literature reviews on adherence have largely focused on obtaining pooled estimates of adherence among pregnant women [8–10]. However, the interpretation of such estimates requires close investigation of underlying methodological approaches partly due to divergent thresholds adopted (e.g., > 50% vs. > 80%). Also, another review of trials in LMICs found that self-report was most common, with great heterogeneity in defining and calculating adherence [6]. This literature review’s primary focus, however, was on pregnant women consuming iron and folic acid (IFA), providing limited implications for how methodological approaches were applied for non-pregnant women or adolescent girls and other micronutrients [6].

Our literature review aimed to provide consolidated evidence by reviewing adherence measurement methodology across various study designs, types of micronutrients, and populations. Reviewing various types of interventions and exploring strengths and weaknesses of each, we hoped to provide a way forward for guiding evidence-based approaches. Specifically, we sought to 1) summarize the characteristics of studies that implemented a micronutrient supplementation intervention for women of reproductive age, 2) synthesize data on adherence definition, measurement, and outcomes, and 3) examine studies that led to improvement in adherence.

## Methods

### Inclusion and exclusion criteria

We included studies that empirically measured adherence to micronutrient supplements among women of reproductive age. Since studies adopted various terms for ‘adherence,’ such as compliance, uptake, and consumption, we considered any micronutrient supplementation behaviors. Studies without health outcomes were included as long as they measured and reported adherence. We considered peer-reviewed original research that evaluated a micronutrient supplementation intervention based on primary data. Studies must have provided enough details about an intervention to describe the objective of a program. Studies that evaluated governmental supplementation programs were included only if they provided sufficient intervention descriptions and collected primary data. Referring to the micronutrient recommendations for women of reproductive age by the WHO and UNICEF and findings from the United Nations International Multiple Micronutrient Antenatal Preparation (UNIMMAP) trials [3, 4, 11], we included studies that focused on iron, folic acid, vitamin A, vitamin E, vitamin B6, vitamin B12, vitamin D, vitamin C, calcium, zinc, and multiple micronutrient supplementation (MMS). Micronutrient supplements must have been in a form of tablet or pill. We included studies that focused on women of reproductive age (15–49 years) and examined interventions relevant to this population. Also, studies were included if they were conducted in LMICs per the World Bank categorization of lending groups in 2022 [12]. Given the possibility that adherence measurement reflects recent changes in the health system, programmatic issues, and technology, we limited to studies conducted between January 2010 and July 2022.

We excluded studies that did not measure any supplementation-related behavioral outcomes among women or those that relied on secondary data to assess adherence. Dietary consumption that did not involve tablets or pills were not considered. Our focus was on adherence measurement methodologies; therefore, studies were eligible even if they did not report health outcomes. However, efficacy trials centered on the biological effects of supplementation were excluded, as this review prioritized adherence measurement approaches within public health interventions implemented in real-world settings. We excluded studies that were designed for women not of reproductive age or those with specific health conditions (e.g., women with obesity) not directly associated with micronutrient deficiencies. The inclusion and exclusion criteria were finalized among a team of four researchers (BAK, AL, RR, and AP).

### Search strategy and methods

The literature search was conducted on PubMed, Scopus, and Embase on July 20, 2022. A search strategy was developed to include the following four key concepts: “micronutrients,” “women of reproductive age,” “supplementation behavior,” and “LMICs.” The concepts and search terms were finalized in April 2022 upon agreement among a team of four researchers (BAK, AL, RR, and AP) and in consultation with a librarian. Search terms for LMICs were adapted from the librarian’s predeveloped list for PubMed. The final search strategy was applied across all databases. An example of our search strategy used in PubMed is provided as a supplementary material(see Additional file 1). All articles retrieved were transferred to Covidence for screening.

### Screening procedure

**Fig. 1:**
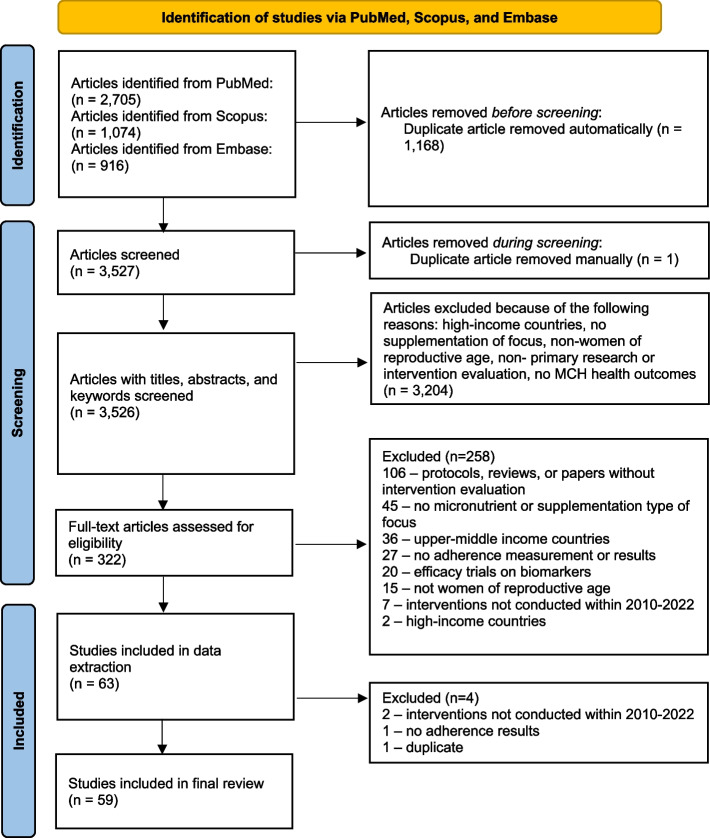
PRISMA flow diagram for a systematic review of adherence to micronutrient supplementation in low- and middle-income countries

A total of 3,527 articles were retrieved after automatically removing duplicates from the software (Fig. 1). Title and abstract screening was conducted on Covidence from April to September 2022. Two independent reviewers (BAK and AL) screened the articles based on the eligibility criteria. Conflicts were resolved by a third study team member (AP). From this screening process, 3,204 articles were excluded as the articles did not meet the inclusion criteria. Full-text screening of 322 articles was conducted from September to November 2022 by two independent reviewers (BAK and AL). Conflicts were resolved by a third study team member (AP). Sixty-three articles entered the data extraction process, during which 4 additional articles were excluded. In total, 59 articles were included for data extraction and analysis.

### Data extraction and quality assurance

A data extraction template was developed and finalized using Covidence. The template was intended to capture study characteristics, including study design, country, population, intervention setting, adherence measurement and outcomes, intervention strategies, and health outcomes. A data extraction guideline was developed by a researcher (BAK) and finalized upon agreement among a team of four (BAK, JB, OO, and AL). Data extraction was conducted from November 2022 to January 2023 by the same four researchers. To ensure reliability of data extraction, 20% of included articles(12 articles) were extracted in the beginning by two independent researchers in pair to reach consensus on the extraction scheme. A third reviewer not assigned to respective articles resolved disagreement. Throughout the data extraction process, a weekly meeting was held to discuss the progress and reach consensus if necessary. As a final step, spot checks of randomly selected articles were conducted to ensure accurate data entry.

### Risk of bias appraisal

Four researchers (BAK, JB, OO, and AL) conducted the quality assessment in pairs and resolved conflicts through regular discussion. The quality of included articles was assessed using the Cochrane tools [13]. We used the “Risk of bias in randomized trials(RoB2)” tool for randomized controlled trials and the 2021 version of the “RoB2 for cluster-randomized trials” tool for cluster randomized controlled trials. The assessment criteria of the tools are based on random sequence generation, deviations, outcomes (supplementation adherence) data and measurement, and selective outcome reporting. All other non-randomized studies were analyzed using the “Risk of bias in non-randomized studies—of interventions(ROBINS-I)” tool. The tool additionally assesses for biases stemming from confounding, participant selection, and classification of interventions.

### Data analysis

Data analysis was conducted by one researcher (BAK) from March to July 2023. We conducted the analysis based on information about study characteristics, adherence definition, adherence measurement methods, adherence outcomes, and intervention type. If adherence was not defined, we considered reporting cutoffs as proxy and indicated accordingly. Furthermore, during analysis, we observed distinct methodological patterns between programs that sought to improve adherence through intervention strategies (e.g., nutritional education) and those that relied solely on supplement provision. Accordingly, we categorized studies into behavioral and non-behavioral groups. For adherence outcomes, we applied each study’s own standards of statistical significance to determine whether the intervention improved adherence. Studies that reported adherence prevalence without statistical testing (e.g., before-and-after prevalence without significance testing) were excluded from this analysis. We then examined patterns among interventions that demonstrated statistically significant improvements in adherence.

## Results

### Study characteristics

The selected 59 studies include 52 unique projects across 19 countries in 4 different global regions(Table 1): Sub-Saharan Africa(39%), South Asia(35%), East Asia and Pacific(21%), and the Middle East(6%). Forty-two(81%) of the included projects were from lower-middle-income countries. The countries most represented were India(*n* = 12, 23%), Indonesia(*n* = 8, 15%), and Bangladesh(*n* = 4, 8%). Twenty-eight projects(54%) were randomized-controlled trials (RCTs), followed by quasi-experimental studies(31%) and cross-sectional studies(10%). Most projects were conducted for pregnant women(51%) or women who recently delivered(18%). The most frequently tested micronutrient supplements across study groups were IFA(43%) followed by iron(15%) and calcium(13%).

**Table 1:** Characteristics of unique micronutrient supplementation projects

Author, year	Country, setting^a^	Study design^b^	Intervention setting^c^	Study objectives^b^	Population characteristics^b^	Total sample size^b,d^
Unique non-behavioral projects (n = 14)						
Nguyen 2016 [14], Ramakrishnan 2016 [15], and Gonzalez-Casanova 2017 [16]	Vietnam (LM), rural	RCT	Home	To evaluate weekly IFA or multiple micronutrient (MM) supplements compared to only folic acid (FA) improves iron status and anemia during pregnancy and early postpartum	Currently married women aged 18–40 years planning on becoming pregnant in the next year, from 20 communes Thai Nguyen province	5011
Abioye 2016 [17]	Tanzania (LM), urban	Cohort Study	Health clinic	The study aims to assess how hematologic biomarkers respond to iron supplementation and identify predictors of this response in iron-deficient pregnant women	Pregnant women in their first or second pregnancy and iron-deficient	600
Vanobberghen 2021 [18]	Tanzania (LM), rural	RCT	Home	To assess the efficacy and safety of intravenous ferric carboxymaltose versus oral iron substitution following childbirth in women with iron deficiency anemia in Tanzania	Women close to delivery with an iron deficiency and living close to the hospital who agreed to attend scheduled follow-up visits	230
Taneja 2021 [19]	India (LM), urban	RCT	Home	To test the efficacy of a nutritional intervention to improve the dietary adequacy of mothers during lactation and improve the growth of their infants	Infants within 7 d of birth and their lactating mothers	816 mother-infant dyads
Karakochuk 2017 [20]	Cambodia (LM), rural	RCT	Community, Home	To assess the effect of daily oral iron with or without multiple micronutrients (MMNs) on hemoglobin concentration in nonpregnant women with anemia	Anemic non-pregnant women aged 18–45 y with a hemoglobin concentration less than 117 g/L	809
James 2019 [21]	Gambia (L), rural	RCT	Health clinic, Home	To test the efficacy of a nutritional supplement that improves one-carbon-related nutrient status by reducing plasma homocysteine	Premenopausal women aged 18–45 years, non-pregnant with no plan to conceive in the next coming months, no plans to travel, and no current illness or chronic health problem	298
Hofmeyr 2019 [22]	Zimbabwe(LM), urban	RCT	Health clinic, Community, Home	To test the hypothesis that calcium supplementation before and in early pregnancy (up to 20 weeks' gestation) prevents the development of pre-eclampsia	Parous women with pregnancy intention and a history of pre-eclampsia or eclampsia during pregnancy	1355
Hanieh 2013 [23]	Vietnam (LM), rural	RCT	Health clinic, Home	To compare the effect of intermittent antenatal iron supplementation with daily iron supplementation on maternal and infant outcomes	Residents of trial commune, older than 16 years old with a confirmed pregnancy at 16 w gestation	1258
Gunaratna 2015 [24]	Tanzania (LM), rural	RCT	Home	To assess the efficacy of pre-pregnancy supplementation with iron and multivitamins to reduce the prevalence of anemia during the periconceptional period	Non-pregnant women aged 15–29 planning to stay in the study area for 6 months	802
Goldberg 2013 [25]	Gambia (LM), rural	RCT	Health clinic, Home	To test the effects on blood pressure (BP) of calcium carbonate supplementation (1500 mg Ca/d) in pregnant, rural Gambian women	Pregnant women	662
Duggan 2014 [26]	India (LM), urban	RCT	Health clinic, Home	To evaluate if daily oral vitamin B-12 supplementation during pregnancy increases maternal and infant measures of B-12 status	Pregnant women aged 18 and older who presented for prenatal care before or at 14 weeks gestation	366
Bharti 2015 [27]	India (LM), rural	RCT	Community	To evaluate the efficacy of directly observed home-based daily iron therapy on the prevalence of anemia	All anemic rural women and adolescent girls aged 13 years and older	1059
Bah 2019 [28]	Gambia (L), rural	RCT	Health clinic	To test a hepcidin-guided screen-and-treat approach can achieve equivalent efficacy to universal administration, but with lower exposure to iron	Women aged 18–45 years at between 14 and 22 weeks of gestation	498
Adaji 2019 [29]	Nigeria (LM), urban	RCT	Health clinic	To compare the effectiveness of the once versus twice daily doses of ferrous sulphate in the prevention of iron deficiency anaemia in pregnancy	Consecutive pregnant women who presented for antenatal booking during the study period at gestational age of 14–24 weeks and gave informed consent	164
Unique behavioral projects (n = 38)						
Morteza 2017 [30]	Iran (LM), not specified	Quasi-experimental study	Community	To determine the effect of the classes of the pregnancy period preparation on the nutritional behavior of the postpartum period	Pregnant Persian women between 18–24 weeks of pregnancy	230
Sedlander 2022 [31] and Ganjoo 2022 [32]	India (LM), rural	RCT	Community	To examine the effect of the intervention at midline to understand which components of the RANI intervention affect uptake	Women between the ages of 15 and 49 who spoke Odiya and planned to live in the data collection area for the next year	4110
Bilimale 2010 [33]	India (LM), rural	RCT	Home	To document the effect on adherence of directly observing patients taking iron therapy of pregnant women	Pregnant women	113
Anitasari 2017 [34]	Indonesia (LM), urban	Quasi-experimental study	Community	To determine if the use of media such as leaflets and short message service (SMS) reminders to improve compliance	Pregnant women from community health clinics who can read and own a cell phone between 14–32 weeks gestational age	74
Heryadi 2017 [35]	Indonesia (LM), urban	RCT	Health clinic	To provide knowledge about anemia during pregnancy, increase adherence to tablets, and improve consumption patterns through counseling	Pregnant women who received ANC at the CHC within the study districts and received supplements through the government program	192
Rukmaini 2018 [36]	Indonesia (LM), rural	Quasi-experimental study	Community, Home	To assess the effect of mobile control application on the compliance of ferrum tablets consumption among pregnant women	Women in the first or second trimester of a singleton pregnancy who are able to use phone applications	86
Surtimanah 2019 [37]	Indonesia (LM), urban	Quasi-experimental study	Health clinic	To determine if family support altered the amount of iron tablets consumed between two groups	All pregnant women in Sukasari community health center who have received their first prenatal care visit and 30 iron tablets for a month	60
Nahrisah 2020 [38]	Indonesia (LM), Not specified	Quasi-experimental study	Home	To determine the effect of individual education through a pictorial handbook on anemia in conjunction with counseling	Pregnant women with hemoglobin levels below11 g/dL, aged 20 years or above, free from obstetrical complications	158
Wulandari 2022 [39]	Indonesia (LM), urban	Quasi-experimental study	Health clinic	To determine the effectiveness of the mobile-health interactive message on the postpartum care behavior of mothers and their husbands	Pregnant women giving birth in the city between 28–34 weeks living with their husband with Whatsapp access	46
Ariyani 2022 [40]	Indonesia (LM), not specified	RCT	Health clinic	To determine the effect of a web application-based antenatal education program using an SCT model on HPLP II scores, compliance, and readiness for childbirth	Healthy pregnant women with parity 1 to 4	193
Walia 2020 [41]	India (LM), rural	Cross-sectional study	Community	To assess whether the behavior change communication intervention improved maternal behaviors among self-help group members	SHG members who gave birth to a live infant in the past year	1204
Shivalli 2015 [42]	India (LM), rural	Quasi-experimental study	Community, Home	To examine the effectiveness of TIPs on dietary and iron-folate tablet intake during pregnancy	Pregnant women in 13–28 weeks of gestation	98
Kamau 2020 [43]	Kenya (LM), rural	Quasi-experimental study	Community	To determine the effect of a community-based approach of IFAS distribution on compliance	Pregnant women aged 18–49 years and below 33 weeks in their pregnancy gestation	340
Gamboa 2020 [44]	Indonesia (LM), not specified	Cross-sectional study	Community	To understand how participation in the IPC activities impacted maternal knowledge, attitude, intention, and consumption	Women of reproductive age living in the sample village clusters	766
Compaore 2018 [45]	Burkina Faso (L), rural	RCT	Home	To explore factors affecting adolescent adherence to weekly iron and/or folic acid supplements in a setting of low secondary school attendance	Non- pregnant women aged 15–24 years living in the 30 villages in the study districts	1959
Byamugisha 2022 [46]	Uganda (L), urban	RCT	Health clinic	To determine the effect of dispensing blister and loose packaged IFA pills on adherence	Pregnant women eligible for IFA supplementation in their second trimester up to 28 weeks	952
Noronha 2013 [47]	India (LM), urban and rural	Quasi-experimental study	Health clinic	To determine the effectiveness of a health information package (HIP) for anemic pregnant women	Pregnant women who attended the antenatal clinic below 20 weeks of gestation	193
Khorshid 2014 [48]	Iran (LM), rural	RCT	Health clinic, Home	To evaluate the effect of SMS on compliance with iron supplementation among pregnant women	Women pregnant with a singleton fetus between 14–16 weeks gestational age with a mobile phone	116
Harding 2017 [49]	Bangladesh (LM), rural	RCT	Community, Home	To evaluate sustained adherence to LNS and how it compares with adherence to IFA and determine programmatic and individual factors associated with adherence	Pregnant women at or before 20 weeks gestation planning to live in the area for 3 years	405
Ahamed 2018 [50]	India (LM), rural	RCT	Community, Home	To estimate the reduction in the prevalence of anemia, improvement in iron status, and to compare the compliance to oral iron supplementation during pregnancy	Pregnant women planning to live in the area for their second trimester and registered for antenatal care at the subcenter	400
Kung’u 2018 [51]	Ethiopia, Kenya, and Senegal, not specified	Quasi-experimental study	Community	To demonstrate how proven nutrition interventions could be integrated into health programs to improve knowledge and practices	Mothers with children 0–11 months in the study regions	997 (Ethiopia), 464 (Kenya), 1444 (Senegal)
Ouedraogo 2019 [52]	Niger (L), rural	Quasi-experimental study	Community	To assess the impact of the programmatic intervention on: (1) utilization of ANC, (2) adherence to daily IFA supplementation, (3) prevalence of adequate gestational weight gain	Pregnant women in the selected villages planning to stay in the study region	2307
Hazra 2020 [53]	India (LM), rural	Quasi-experimental study	Community	To assess the effects of health behavior change interventions through women self-help groups on maternal and newborn health	Married women of reproductive age that gave birth in the last 12 months	4615
Kurzawa 2021 [54]	Bangladesh (LM), rural	Quasi-experimental study	Health clinic, Community	To examine the effects of capacity building of frontline health care workers on IFA consumption and adherence by pregnant women	Women who gave birth within the past 6 months	Unclear
Nguyen 2021 [55]	India (LM), rural	RCT	Health clinic, Community	To compare nutrition-intensified ANC (I-ANC) with standard ANC (S-ANC) on coverage of nutrition interventions and maternal nutrition practices	Recently delivered women with children less than 6 months of age or pregnant women in the second and third trimesters of pregnancy	2460
Sharma 2016 [56]	Nepal (LM), rural	Cross-sectional study	Health clinic, Community	To evaluate an intervention to promote maternal health among young women in Nepal	Women aged 15–49 with at least one child under 2	1236
Nguyen 2018a [57], Todd 2019 [58], Nguyen 2017 [59], and Nguyen 2018b [60]	Bangladesh (LM), rural	RCT	Health clinic, Community, Home	To evaluate the effect of providing nutrition-focused MNCH compared with standard MNCH on coverage of nutrition interventions, maternal dietary diversity, micronutrient supplement intake, and early breastfeeding practices	Recently delivered women with children less than 6 months of age	2000
Brasington 2016 [61]	Egypt (LM), not specified	Quasi-experimental study	Community, Home	To assess the impact of SMART activities on knowledge and behaviors related to pregnancy and newborn care among mothers of young children	Mothers who had children less than one-year-old	3445
Thapa 2016 [62]	Nepal (LM), rural	Cross-sectional study	Health clinic	To assess the coverage, compliance, acceptability, and feasibility of the intervention	Recently delivered women within past 6 months	1240
Srivastava 2019 [63]	India (LM), urban	RCT	Health clinic, Home	To test whether the compliance to capsule formulation would be better than tablet formulation for oral iron supplementation among pregnant Indian women	Pregnant women 18 and older with gestational age > 12 weeks who had not consumed any iron supplement prior to enrollment	204
Riang'a 2020 [64]	Kenya (LM), rural	Cross-sectional study	Health clinic	To determine the degree to which the nutritional counselling and IFAs guidelines during pregnancy have been implemented as intended	Pregnant mothers who had at least one previous ANC visit at a health facility and mothers who had delivered within 1 month	188
Omotayo 2017 [65], Omotayo 2018a [66], Omotayo 2018b [67], and Martin 2017 [68]	Kenya (LM), rural	RCT	Health clinic, Community, Home	To employ the trials of improved practices (TIPs) approach and explore adoption and acceptability of the WHO recommendations among pregnant women	Pregnant women aged 15 and older from 16–30 weeks planning to stay in the area for at least 6 weeks	1036
Nwaru 2015 [69]	Mozambique (L), urban	RCT	Health clinic	To examine the extent of participants adherence to IFA tablets	Women who were not at high obstetric risk and women aged 18 years and older	4326
Klemm 2020 [70]	Ethiopia (L), rural	RCT	Home	To identify adherence strategies and integrate calcium into antenatal IFA supplementation programming	Healthy pregnant women aged 16 or older, between 12 and 28 weeks	48
Clermont 2018 [71]	Niger (L), rural	RCT	Community, Home	To examine the factors influencing acceptability and utilization of these three supplements	Pregnant women less than 30 weeks gestation at the time of enrollment	53
Baxter 2014 [72]	Bangladesh (LM), urban	Discrete-choice experimental design	Community, Home	to evaluate preference and acceptability of 4 different options for delivering prenatal calcium to pregnant women	Pregnant women aged 18 to 39 y and a gestational age of 13 to 30 weeks	132

^a^L: low-income country, LM: lower-middle-income country

^b^For studies under the same project, information available for the parent study is provided

^c^Places where most interventions activities occurred

^d^The number of women that received interventions

### Adherence definition

We considered studies that were under the same project as constituting separate studies, given that adherence cutoffs and outcomes tended to be distinctively reported. Therefore, 59 included studies(16 non-behavioral and 43 behavioral studies) were analyzed(Table 2). In this section, we followed studies’ description on adherence definition or their use of a designated cutoff to dichotomize women who were adherent and nonadherent in reporting. Notably, adherence was frequently defined as the proportion of provided supplements consumed, with a common threshold set at 80% or higher across the 16 non-behavioral studies [14–29]. A study involving MMS, IFA, and folic acid supplementation groups demonstrated that 78% of overall women across the study groups consumed over 80% of provided supplements [14]. Another study assessed adherence to monthly IFA supplementation ANC and reported 40% compliance at 80% or more [17]. However, none of the non-behavioral studies provided justification on selected thresholds for adherence definition.

**Table 2:** Definition, measurement, and outcomes of adherence to micronutrient supplementation

Author, year	Micronutrient^a^	Supplement provision	Adherence definition^b^	Adherence measurement	Measurement frequencies	Adherence monitoring^c^	Adherence outcomes^d^	Health outcomes^d^
Non-behavioral studies (N = 16)								
Nguyen 2016	MMS (I_1_), IFA (I_2_), FA (C)	Every two weeks	> 80% of provided supplements	Pill count (out-of-clinic); direct observation by health worker	> 5	Y (direct observation every two weeks at home visit)	78% of the overall women consumed > 80%; no differences in compliance by intervention group both before conception (weekly) and during pregnancy (daily). No statistical significance available	No differences in anemia across groups
Abioye 2016 [17]	IFA (I)	Monthly supply of IFA during ANC	≥ 80% of days; duration of supplementation > 90 days	Pill count (in-clinic)	5	Y (In-clinic pill count at monthly visit)	239 women (40%) compliance ≥ 80%, 361 (60%) women compliance < 80%, 321 (54%) women took supplements ≥ 90 days, 279 (46%) women < 90 days. No statistical significance available	**Within the whole population, hemoglobin improved baseline (101 g/L) to delivery (117 g/L), *p* <.0001**
Vanobberghen 2021 [18]	Intravenous iron (I_1_), oral IFA (I_2_)	1500–2000 mg in two doses at least 7 days apart depending on body weight	≥ 90% for oral iron	Pill count (out-of-clinic)	5	Y (Consumption log, pill count at each visit)	Oral iron: 80(79%) had 90% adherence as determined at 6 weeks. 47 (47%) women 90% adherence 3 months after. No statistical significance available	51% of participants in the oral iron group normalized hemoglobin, but lower than intravenous iron
Taneja 2021 [19]	MMS (I), Placebo (C)	MMS tablets for 1 month during home visit	N/A	Pill count (out-of-clinic)	> 5	Y (Pill count during a monthly home visit)	Mean number of daily consumption = 146.54. The proportion of mothers who consumed a tablet for > 75% of the days was 78.7%; No statistical significance available	No difference b/w groups in length for age (infant at 6 months old) **Hemoglobin: 11.99 ¬ ± 1.16, *p* <.001** **Anemia rates 39.6%, *p* <.002**
Ramakrishnan 2016 [15]	MMS (I_1_), IFA (I_2_), FA (C)	Every two weeks	> 90%	Pill count (out-of-clinic); direct observation by health worker	> 5	Y (direct observation)	Compliance was > 90% for both preconception and prenatal supplements; Compliance did not differ by study group; No statistical significance available	No differences in anemia or birth outcomes across groups
Karakochuk 2017 [20]	Iron only (I_1_), MMS (I_2_), Iron + MMS (I_3_), Placebo (C)	Monthly supply of 1 bottle (30 capsules)	≥ 80% of capsule consumption	Pill count (in-clinic) every month	3	Y (Pill count at a village center)	**I**_**1**_**:86%, I**_**2**_**:89%, I**_**3**_**:78% (lowest among the intervention groups with highest side effects, and C: 88% of women were adherent, (chi-square, *p* < 0.05)**	**Anemia prevalence rates after intervention were I**_**1**_**:42%, I**_**2**_**: 58%, I**_**3**_**: 39%, and C: 58%; Iron significantly increased hemoglobin at 12 wk, but MMSs did not**
James 2019 [21]	Vitamin B (I_1_), MMS (I_2_), Placebo (C)	Weekly packs of supplements during home visit	> 80% of the number of supplementation cards	Observation by health workers; Consumption card cross-checking with the number of remaining pills	> 5	Y (Health worker report; Weekly submission of consumption card at home visit)	Both study arms showed high adherence. I_1_: 98.8%, I_2:_ 97.8% (*p* = 0.23)	There was no effect of either intervention on blood pressure or pulse compared with the control at endline
Hofmeyr 2019 [22]	Calcium (I), Placebo (C)	One tablet daily < 20 weeks' gestation. After 20 weeks, provided supplements that last 12 weeks	> 80% (Reporting was done for both > 80% and > 50%)	Pill count (in-clinic)	> 5	Y (Health worker monitoring and visits; number of returned tablets)	Compliance > 80% From randomisation up to the last visit before pregnancy = 47% (*p* = 0.817, between groups); From last visit before pregnancy to the 20-week visit = 53%, (*p* = 0.563, between groups) **Compliance > 50%** **From randomisation up to the last visit before pregnancy = 81%,(*p* = 0.011, between groups)**; From last visit before pregnancy to the 20-week visit = 77%, (*p* = 0.292, between groups)	Pre-eclampsia (primary outcome) (23%) *p* = 0.121 (between groups) Gestational hypertension (66%), *p* = 0.296 (between groups) Stillbirth (9%), *p* = 0.316 (between groups)
Hanieh 2013 [23]	IFA daily (I_1_), IFA twice/week (I_2_), MMS twice/week (I_3_)	Provided supplements during home visit every 6 weeks	Not defined, no cut-off reported	Health worker report every 6th week; Self-report in-person interview	5	Y (Self-report in-person interview)	**Daily IFA (reference): The median level of adherence 91%; Twice IFA: 96%, significantly higher than daily IFA (*p* =.01); Twice MMN: 85%, significantly lower than daily IFA (*p* <.001)**	Daily IFA (reference): Hemoglobin, mean birth weigh were not statistically different among the groups **Twice IFA: Hemoglobin OR 0.03, *p* = 0.98; Infant cognitive development higher than daily IFA (MD 1.89. *p* <.03)** Twice MMN: Hemoglobin OR −1.07, *p* = 0.40; Infant cognitive development (MD 0.79. *p* = 0.31)
Gunaratna 2015 [24]	Folic acid only (I_1_), IFA (I_2_), Folic acid + multivitamins + iron (I_3_)	Monthly provision during home visit	≥ 80% of days taking supplements	Pill count (out-of-clinic); Self-report in-person interview	5	Y (Self-report and pill count monthly)	FA arm: 82% compliance (reference), IFA arm: 84%, *p* =.40 compared to the FA arm, FA + MV arm: 83%, *p* =.67 compared to the FA arm	FA arm: RR for anemia 1 (ref) IFA arm: RR for anemia 0.91, *p* = 0.40 compared to the FA arm FA + MV arm: RR for anemia 0.86, *p* = 0.18 compared to the FA arm
Gonzalez-Casanova 2017 [16]	MMS (I_1_), IFA (I_2_), FA (C)	Every two weeks	> 80% of provided supplements	Pill count (out-of-clinic); direct observation by health worker	> 5	Y (Direct observation)	Across study groups, 78% of the women consumed > 80%; no differences by groups	No differences in anemia across groups
Goldberg 2013 [25]	Calcium (I), Placebo (C)	Provided 3 supplements weekly at ANC	Not defined, no cut-off reported	Pill count (in-clinic); Observation by health workers	4	Y (Weekly checking of a ticket and tablet count)	I: Mean compliance 97%; C: 97%, No statistical significance available	Birth weight P20 = SD ± mean = 55.1 ¬ ± 6.9 (*p*-value = 0.7; between groups)
Duggan 2014 [26]	Vitamin B12 (I), Placebo (C)	Provided bottles containing 40 tablets by pharmacists	Not defined, no cut-off reported	Pill count (in-clinic)	> 5	Y (Monthly measured by research nurses counting unused supplements)	I: Mean compliance rate 69%, C: 70% (*p* = 0.74)	Incidence of delivering an infant with intrauterine growth retardation was 33 of 131 (25%) (*p* = 0.11)
Bharti 2015 [27]	Directly observed IFA (I), self-treatment IFA (C)	Daily supervision of iron therapy by volunteers for 90 days	Not defined, no cut-off reported	Observation by health workers	> 5	Y (Daily supervision)	**I: 93%, C: 60% (*p* <.0001)**	**Relative risk of anemia: 16.8%** ***P* <.0001**
Bah 2019 [28]	MMS (I_1_), screen-and-treat 60 mg MMS(I_2_), screen-and-treat 30 mg MMS(I_3_)	Provided 7 supplements per week	Not defined, no cut-off reported	Pill count (in-clinic)	> 5	Y (Pill count, direct observation)	86% in all groups. No statistical significance available	**I**_**1**_**:Anemia reduced from 58% (day 0) to 45% (day 84)** I_2_: 60 mg group: Anemia rose from 52% (day 0) to 57% (day 84) I_3_: Anemia rose from 53% (day 0) to 59% (day 84)
Adaji 2019 [29]	Once iron (I_1_), twice iron (I_2_)	Provided daily or twice daily supply of iron with folic acid daily at ANC	≥ 65%	Pill count (in-clinic), self-report	2	Y (Counting blister packets at each ANC visit)	**I**_**1**_**: 75%, I**_**2**_**: 55% (*p* < 0.01)**	Birth weight (mean ± SD) = 69, 3.2 ± 0.4 (*P* = 0.986; between groups); Low birth weight (< 2.5 kg) = 14, 1.9 ± 0.4 (p-value = 0.928, between groups)
Behavioral studies (N = 43)								
Morteza 2017 [30]	Iron (I), iron (C)	Unclear (ANC distribution)	Not defined. Duration of iron supplementation 0, 1, 2, 3, 5 month	Self-reported questionnaire	1	N	**I (pregnancy preparation class): 47% of women took iron 3 months post-partum** **C (usual care): 16% of women in the control group (*p* <.001)**	N/A
Sedlander 2022 [31]	IFA (I), IFA (C)	Unclear (ANC distribution)	Not defined, adherence was reported binary	Self-reported questionnaire	1	N	**I (community-based education): 32%** **C (usual care):3% (*p* < 0.001)**	Hemoglobin Levels: I: 11.7%, C: 11.5% (*p* = 0.28)
Bilimale 2010 [33]	IFA (I); IFA (C)	Provided iron tablets for 100 days across three intervals	Not defined. Reporting was done for both > 80% and > 50%	Pill count (out-of-clinic); Observation by community member	3	Y (I: daily direct observation)	**I (direct observer with counseling): 2nd visit 78.48% vs. C (counseling): 49.22% (*p* <.001)** **I: 3rd visit 79.13% vs. C: 52.75% (*p* <.001)** **I: 4th visit 76.44% vs. C: 53.87% (*p* <.001)**	N/A
Anitasari 2017 [34]	Iron (I), iron (C)	Government distributes 90 day supply of supplements	Not defined. Reporting based on MMAS-8 Questionnaire (A score of 0 = compliance, 1–2 = moderate compliance, and > 2 = non-compliance)	Self-reported questionnaire	2	N	**I (leaflets) & C (SMS): Decreased percentage of respondents in the non-compliance category (*p* <.05) and increase in percentage of respondents in the medium and obedient compliance categories**	I (leaflet): 19 respondents had an increased hemoglobin levels (average of 0.6 g/dl) C (SMS): 17 respondents had an increased hemoglobin level (average of 1.1 g/dl). Not statistically significant
Heryadi 2017 [35]	Iron and calcium (I), iron and calcium (C)	Unclear	Not defined. Reporting by the number of tablets (0–15, 16–30, 31–45, 45–60 tablets)	Pill count (in-clinic)	2	N	**I (counseling): Calcium and iron consumption 0.38¬ ± 0.49 (pre); 1.0¬ ± 0.0 (post) (*p* <.001)** **C (no counseling) (*p* = 1.00)**	**I: Hemoglobin 10.39¬ ± 1.24 (pre); 11.52¬ ± 0.92 (post), (*p* <.001)**
Rukmaini 2018 [36]	Iron (I)	Provided 90 tablets of oral iron during ANC visit	Not defined. No cut-off reported	Self-reported questionnaire (10 items); mobile control application used by women	2	N	**I (mobile app): Pretest: 33.7%, Posttest: 72.1%** **(*p* <.01)**	N/A
Surtimanah 2019 [37]	Iron (I_1_), iron (I_2_)	Provided 30 day supply during ANC visit	Not defined. Reporting was done for the percentage of pregnant women who consumed 30 iron tablets for a month	Self-report in-person interview; Tablet compliance card	1	N	**I**_**1**_** (Pregnant women class group): 60%, I**_**2**_** (midwife counseling group): 93.30% (*p* < 0.01)**	N/A
Nahrisah 2020 [38]	IFA (I), IFA (C)	Provided IFA during ANC visit	Not defined, no cut-off reported	Self-report in-person interview	2	N	**I (education and counseling) Mean number of IFA intake 55.5, higher than control group (usual care) (*p* <.001)**	**All anemic pregnant women in the intervention group recovered from anemia during the third trimester of pregnancy** **Birth weight (mean): 3324 g, higher than the control group (*p* <.01)**
Wulandari 2022 [39]	Iron and vitamin A (I), iron and vitamin A (C)	Unclear	Not defined. Consumption of vitamin A and iron tablets was reported binary	Self-reported questionnaire; Observation by health workers	3	N	**I (mHealth): The chance of consuming iron tablets was 2.64 times higher than the control group (*p* <.01)**	N/A
Ariyani 2022 [40]	Iron (I), iron (C)	Thirty iron tablets (60 mg) were administered at each ANC	≥ 80% of 90 days	Pill count (in-clinic)	2	N	I (web-based ANC education): 31% increase in compliance with iron tablet consumption in treatment (p <.001) C (regular ANC): 12.2% increase (*p* <.05) No difference in compliance with iron tablet consumption was found between the treatment (95.8%) and control (91.9%) groups	N/A
Walia 2020 [41]	IFA (I), IFA (C)	Unclear (provided tablets at ANC)	Not defined. Consumption of tablets at least 100 days was reported binary	Self-report in-person interview	1	N	**I (self-help group with behavior change messaging): 14.9%** **C (self-help group): 7.5%** **Average effect of exposure among the exposed: 7.4 (*p* <.05)**	N/A
Shivalli 2015 [42]	IFA (I), IFA (C)	Unclear (84% of women either received or purchased IFA)	Not defined. No cut-off reported	Pill count (out-of-clinic); Self-report in-person interview	2	N	I (Trials of Improved Practices): 85% of pregnant women were compliant. C (usual care): 38% were compliant. No statistical significance available	**I: The prevalence of anemia, reduced by half (65.9% to 31.1%) C: increased by 2.4% (*p* <.01)** **I: Mean hemoglobin were 11.5¬ ± 1.24 g/dl C:10.37 ¬ ± 1.38 g/dl (*p* <.01)**
Kamau 2020 [43]	IFA (I), IFA (C)	Provided 7 capsules brought to the home weekly (I)	≥ 70% of doses (5 tablets per week)	Pill count (out-of-clinic)	2	Y (Pill count at home)	**I (Community IFA distribution and counseling): Compliance increased by 8% (from 63.8% to 71.4%) (*p* < 0.05)** **C (Hospital IFA distribution): compliance increased by 6% (from 68.5% to 74.3%). No significant DID was found**	N/A
Gamboa 2020 [44]	IFA (I)	Unclear (provided IFA at ANC)	Not defined. Consumption of IFA was reported binary	Self-report survey	1	N	I (interpersonal communication campaign): Consumption 95.7%. No statistical significance available. Exposure to campaign showed 7.35 greater odds of being given or buying IFA (*p* <.0001)	N/A
Compaore 2018 [45]	IFA (I), folic acid (C)	Provided during weekly home visit	Good (> 50 weekly consumption), medium (25–50), poor (< 25) adherence	Health worker report	> 5	Y (observation)	Median adherence was 79% with no difference by trial arm	N/A
Byamugisha 2022 [46]	IFA (I), IFA (C)	Provided 30-day supply of IFA at monthly ANC visit	Definition of adherence varied by time point (4th week: 100% adherence equivalent to 28 out of 30 pills; 8th week: 100% and 90% adherence equivalent to 15 out of 30 pills)	Pill count (in-clinic; Self-report interview	2	Y (Collected blister packs monthly)	I (blister packaging): Week 4: 40.6%, Week 8: 51.9% C (loose packaging): Week 4: 39%, Week 8: 46.8%, both not statistically significant by study arm	**I: Mean hemoglobin Week 4 11.9 + 1.1 g/dl** **C: 11.8 + 1.3 g/dl** **(*p* < 0.05)** Mean Hemoglobin at Week 8 was not significantly different
Noronha 2013 [47]	IFA (I), IFA (C_1_), IFA (C_2_)	Provided IFA to intervention group	Not defined. Cut-offs of full, partial, and non-compliance were reported	Self-reported questionnaire	3	N	I (IFA + education): 42 out of 77 women were compliant, and 25 women were partially compliant C_1_ (IFA supplementation): 20 women were compliant, 41 were partially compliant C_2_ (IFA supplementation): 39 were compliant, 25 were partially compliant. No statistical significance available	**I: the difference in anemia prevalence across compliance groups within group at *p* < 0.001** **Associations between adherence and health outcomes were tested; all groups were significant at *p* <.001**
Khorshid 2014 [48]	Iron (I), iron (C)	Provided 100 iron tablets (3-month supply) at ANC visit	Not defined. Cut-offs of high (63–84%), moderate (42–62%), and low (< 42%) of tablet consumption were reported	Pill count (in-clinic); Health clinic records	1	N	**I (SMS reminders): 94% high, 4% moderate, and 2% low compliance** **C (usual care): 66% high, 18% moderate, and 16% low compliance (*p* <.01)**	I: Hemoglobin reduced from 11.5 g/dL to 11.2 g/dL. C: Hemoglobin reduced from 11.6 g/dL to 11.2 g/dL No difference by study group *p* = 0.96
Harding 2017 [49]	Lipid-based nutrient supplement (I_1_), IFA (I_2_)	Provided supplements at monthly home visit	Not defined. Cut-offs of high (≥ 70%) low (1–69%) and non (0%) adherence were reported	Self-reported questionnaire; Pill count (out-of-clinic) was conducted but excluded from analysis	2	Y (Pill count)	Adherence as recommended did not differ by study group. During pregnancy, high adherence was lower for I_1_ (LNS-PL, 67%) than for I_2_ (IFA, 87%)	N/A
Ganjoo 2022 [32]	IFA (I), IFA (C)	Unclear. (Provided IFA in the community or at ANC visits)	Not defined. Consumption of IFA was reported binary	Self-report in-person interview	2	N	**I (Community education): IFA use increased from 5.4% to 31.7%. Higher than the control group, (*p* <.001)**	N/A
Ahamed 2018 [50]	IFA (I), IFA (C)	Provided IFA monthly by health workers	> 80% of 100 days	Pill count (out-of-clinic); Observation/by health workers	> 5	Y (weekly supervision by ASHA	**I (direct observation): 69.1%** **C (usual care): 60.4% (*p* < 0.01)**	**I: prevalence of anemia decreased 92.9% to 79.9% (*p* < 0.001); Reduction was higher in the intervention group than in the control, but not significant (*p* = 0.219)**
Kung’u 2018 [51]	IFA (I), IFA (C)	Unclear (ANC distribution)	At least 90 days during pregnancy	Self-report interview	2	N	Ethiopia I: 0.5% to 23%, *p* <.001; C: 0.82% to 18%, *p* <.001 Intervention effect *p* =.401 **Kenya** **I: 22% to 36%, *p*** <.001; C: 21% to 18%, ***p*** =.767 Intervention effect ***p* <.05** Senegal I: 53% to 61%, p <.001; C: 80% to 84%, *p* =.096 Intervention effect *p* =.80	N/A
Ouedraogo 2019 [52]	IFA (I)	Unclear (ANC distribution)	Not defined. Reporting based on daily consumption in last 7 days if received any IFA during pregnancy	Self-report survey	2	N	**Daily consumption in the last 7 days increased from 32.8% to 46.4%, *p* <.05**	Anemia prevalence decreased from 79.5% to 78.2%, *p* =.41 (fully adjusted)
Hazra 2020 [53]	IFA (I), IFA (C)	Unclear	Consumption of 100 or more IFA tablets	Self-report survey interview	2	N	Consumption of 100 or more IFA tablets during pregnancy (DID 1.9, p = 0.186)	N/A
Kurzawa 2021 [54]	IFA (I), IFA (C)	Unclear	At least 90 tablets during pregnancy	Self-report survey	2	Y (health management information systems to track reported adherence)	**I: Adherence rate increased 0.355 to 0.840. Adherence 40.35% higher than control group (DID *p* < 0.01)**	5060 total DALYs averted (1108 maternal mortality, 1767 neonatal mortality, 565 anemia, 848 preterm birth, 777 low birth weight)
Nguyen 2021 [55]	IFA and Calcium (I), IFA and Calcium (C)	180 IFA and 360 calcium tablets during pregnancy	Not defined. Consumption of 100 tablets for IFA and calcium was reported	Self-report survey interview	2	Y (monitoring card checked monthly)	**I: Calcium consumption increased from 3.4% to 13.5% (DID 11.5, *p* <.01)** **IFA consumption increased from 8.0% to 26% (DID 9.5,*p* < 0.05); Consumption > 100 tablets was not significant**	N/A
Sharma 2016 [56]	IFA (I), IFA (C)	Unclear (ANC distribution)	Not defined, no cut-off reported	Self-report survey interview	3	N	**I: IFA intake increased from 86.5% to 96%. OR = 1.9, *p* < 0.001** **DID 3.0, *p* <.05**	N/A
Nguyen 2018a [57]	IFA and Calcium (I), IFA and Calcium (C)	Unclear (provided by study)	Not defined, no cut-off reported	Self-report survey	2	Y (monitoring book checked monthly)	**Intervention villages with better training and knowledge had higher IFA consumption compared to control villages, *p* <.01**	N/A
Brasington 2016 [61]	IFA (I), IFA (C)	Unclear (ANC distribution)	At least 90 tablets during pregnancy	Self-report survey interview	2	N	**Adherence in the low and high exposure groups were 15.2% and 40.5%, respectively in Upper Egypt (*p* <.001) and 29.7% and 44.4% in Lower Egypt (*p* <.001)**	N/A
Todd 2019 [58]	IFA and Calcium (I), IFA and Calcium (C)	Unclear (provided by study)	Not defined, no cut-off reported	Health worker record of # of tablets consumed	3	Y (monitoring book checked monthly)	**Mean IFA and calcium supplement intake was** **higher in the intervention areas in all time periods (*p* <.01)**	Reported anemia rates were similar between the 2 groups (5.7%, intervention; 6.5%, control; *p* = 0.83) **Reported postpartum complications were significantly lower among women in the** **intervention group than the control group (33.5% versus 48.2%, *p* < 0.05),**
Thapa 2016 [62]	Calcium (I)	One-time provision of tablets to last for 5 months or until the end of pregnancy (ANC)	Two tablets daily for 150 days or until delivery (full), 90–149 days (partial), < 90 days (short)	Pill count; Self-report survey interview	2	N	Among women who received calcium, 67.3%, 24.1%, and 8.6% reported full, partial, short adherence. No statistical significance available	N/A
Srivastava 2019 [63]	IFA capsule (I), IFA tablet(C)	Blister pack of 30 tablets or capsules replenished at monthly visit	≥ 90% as good compliance, < 90% as poor compliance	Pill count	3	N	Capsule group (1): 22% women, Tablet group (C): 16.8%, not statistically significant	Hemoglobin level in intervention group: 0.79, control group: 0.44, *p* =.112
Riang'a 2020 [64]	IFA (1)	Unclear (iron and folic acid tablets provided at every ANC separately)	Not defined. Finishing the supplements (minimum 30) received during prior ANC visit	Pill count; Self-report interview	1	N	68% women finished iron supplements, and 64% women finished folic acid supplements, No statistical significance available	N/A
Omotayo 2018b [67]	Calcium and IFA for all intervention groups	Provided pills 4 times (provided by study)	Not defined, no cut-off reported	MEMS (recording bottle openings)	3 (obtaining MEMS data)	Y(electronic monitoring)	Consumption after third visit: 540 mg (2-dose group), 860 mg (3-dose group), 430 (4-dose group), No statistical significance available	N/A
Omotayo 2017 [65]	Calcium and IFA (I), Calcium and IFA (C)	Unclear (Prescription for daily 2 Calcium pills and 1 IFA (I), 3 Calcium pills and 1 IFA (C))	Not defined, no cut-off reported	Pill count (estimated % of adherence based on mean daily amount of calcium supplement consumed)	2	Y (pill-taking calendar used at home)	3-dose (I): consumption 1198 mg/d, adherence 79%; 2-dose (C): consumption 810 mg/d, adherence 81%; not statistically significant	N/A
Omotayo 2018a [66]	Calcium and IFA (I), Calcium and IFA (C)	Unclear (Prescription for daily 2 Calcium pills and 1 IFA (I), 3 Calcium pills and 1 IFA (C))	Not defined. Mean daily consumption as a percentage of prescribed daily consumption	Pill count	2	Y (pill-taking calendar used at home)	IFA adherence: 77%, Calcium adherence: 83%, No statistical significance available	N/A
Nwaru 2015 [69]	Routine Fe (I_1_), Selective Fe (I_2_)	Provided 30 tablets of Fe and folic acid to last one month, calculated to last to the next visit	Not defined. ‘Regular’ intake reported at every ANC visit (as opposed to intermittent or non-consumption)	Self-report survey (one item on prior week consumption)	4	N	70.7% women in selective Fe 71.6% in regular Fe groups (1^o^ de Maio health center). 62.6% women in selective Fe 59.4% in regular Fe groups (Machava health center). Not statistically significant for each visit	N/A
Nguyen 2017 [59]	Calcium and IFA (I), Calcium and IFA (C)	Free IFA and calcium tablets during monthly home visit (I), Sale of IFA and calcium tablets and free distribution at government facilities (C)	Not defined, no cut-off reported	Self-report survey interview. Health worker record of tablets consumed in a mother-baby book (available for 88% of mothers)	2	Y (monitoring book checked monthly)	**DID effect estimates for having consumed IFA and calcium supplements during pregnancy were 9.8 and 12.8 pp, between baseline and endline adjusted for clustering effect at the district and subdistrict levels, *p* <.001**	N/A
Nguyen 2018b [60]	Calcium and IFA (I), Calcium and IFA (C)	Unclear (free tablets provided by study)	Not defined, no cut-off reported	Self-report survey interview; Health worker record of self-monitoring card	2	Y (monitoring book checked monthly)	**Improvements were significantly greater in the nutrition-focused MNCH areas, compared with standard MNCH areas, for the consumption of IFA (DID: 46 tablets), calcium supplements (DID: 50 tablets)**	N/A
Martin 2017 [68]	Calcium and IFA (I), Calcium and IFA (C)	Unclear (ANC distribution)	Not defined. Percentage of supplements taken as prescribed. High adherence if > 85% and low adherers if it was $\le$ 85%	Pill count, Self-reports	2	Y (pill-taking calendar used at home)	Most participants were classified as high adherers for calcium [pill count (n = 590), 68.8%; self- report (n = 914), 77.7%] and IFA [pill count (n = 548), 74.8%; self-report (n = 909), 83.7%], No statistical significance available	N/A
Klemm 2020 [70]	4 events of IFA and Calcium (I_1_), 3 events of IFA and Calcium (I_2_), 2 events of IFA and Calcium (I_3_)	Offered a choice of 2 calcium (conventional and chewable) and IFA if women didn’t receive from ANC visits for six weeks	Not defined. Adherence rate based on the number of MEMS events per prescribed doses Reporting threshold: “very high” (90%–100%), “high” (75%–89%), “low” (45%–74%), and “very low” (< 45%)	MEMS and self-reports	3	Y (MEMS)	Adherence rate in assigned regimen (weeks 0–4): 77.1% (4-event group), 83.4% (3-event group), 81.1% (2-event group), not statistically significant Adherence rate in chosen regimen (weeks 4–6): 95.9% (4-event group), 80.1% (3-event group), 73.8% (2-event group), not statistically significant	N/A
Clermont 2018 [71]	IFA (I_1_), MMN (I_2_), LNS (I_3_)	Provided supplements to last 10 days during weekly home visit (IFA: a blister pack of 10 tablets, MMS: a bottle of 40 capsules, LNS: 10 sachets	Correct consumption as 2 days’ worth of supplements above or below the expected number at the time of the spot check. Reporting cutoffs: under-consumption (less than expected) over-consumption (more than expected)	Pill count (unannounced spot checks)	2	N	**Adherence varied significantly across the three supplement types. IFA: 46% correct consumption, MMN tended strongly toward under-consumption (71%) and MQ-LNS tended to be over-consumed (48%), no p-values reported**	N/A
Baxter 2014 [72]	Calcium (I)	Provided a 1-week supply of 4 types of calcium supplements at weekly home visit	Not defined. Selection of a type of supplement was defined as the proportion of days on which each delivery vehicle was selected during the 21-d selection period	Pill count, Self-report	3	Y (self-tracking form)	**The proportion of delivery vehicle selections was significantly higher at home visit days compared with non-home visit days, *P* < 0.001. The selection of conventional tablets increased between weeks 1 and 3, whereas the selection of flavored and unflavored powder decreased during this time, *P* < 0.001**	N/A

^a^I: Intervention group (specified by number if there are more than one intervention group), *C *Control group

^b^If adherence definition is not available, reporting cutoffs are described

^c^Y: Monitoring activities taken place regularly to track supplement consumption during intervention, *N* No monitoring

^d^Significant outcomes are bolded; results without statistical significance test were not considered as significant outcomes; *I* Intervention group, *C* Control group, *DID* Difference-in-difference

Adherence was defined more diversely across behavioral studies [30–72]. One-third of them used a wide variety of definitions, including a range from 70 to 90% of recommended supplementation, more than 90 days during pregnancy, and ordinal categories(e.g., full, partial, short) based on the number of supplements consumed by women. Studies were commonly following national recommendations(e.g., at least 90 days of supplementation) to define adherence; yet, other definitions were seldom informed by scientific evidence. Twenty-four(67%) of behavioral studies did not provide a definition, 13 of which provided reporting cutoffs alternatively. For example, studies in India reported IFA consumption as a binary outcome, categorizing current consumption as being adherent and previous and non-usage as non-adherent [31, 32]. Also, another behavioral study noted the lack of accepted cutoffs for antenatal supplementation and selected 85% as the study’s standard based on mean adherence to both calcium(86%) and IFA(88%) [68]. Eleven behavioral studies provided neither a definition nor cutoffs, but used continuous adherence measures, instead.

### Adherence measurement

**Fig. 2:**
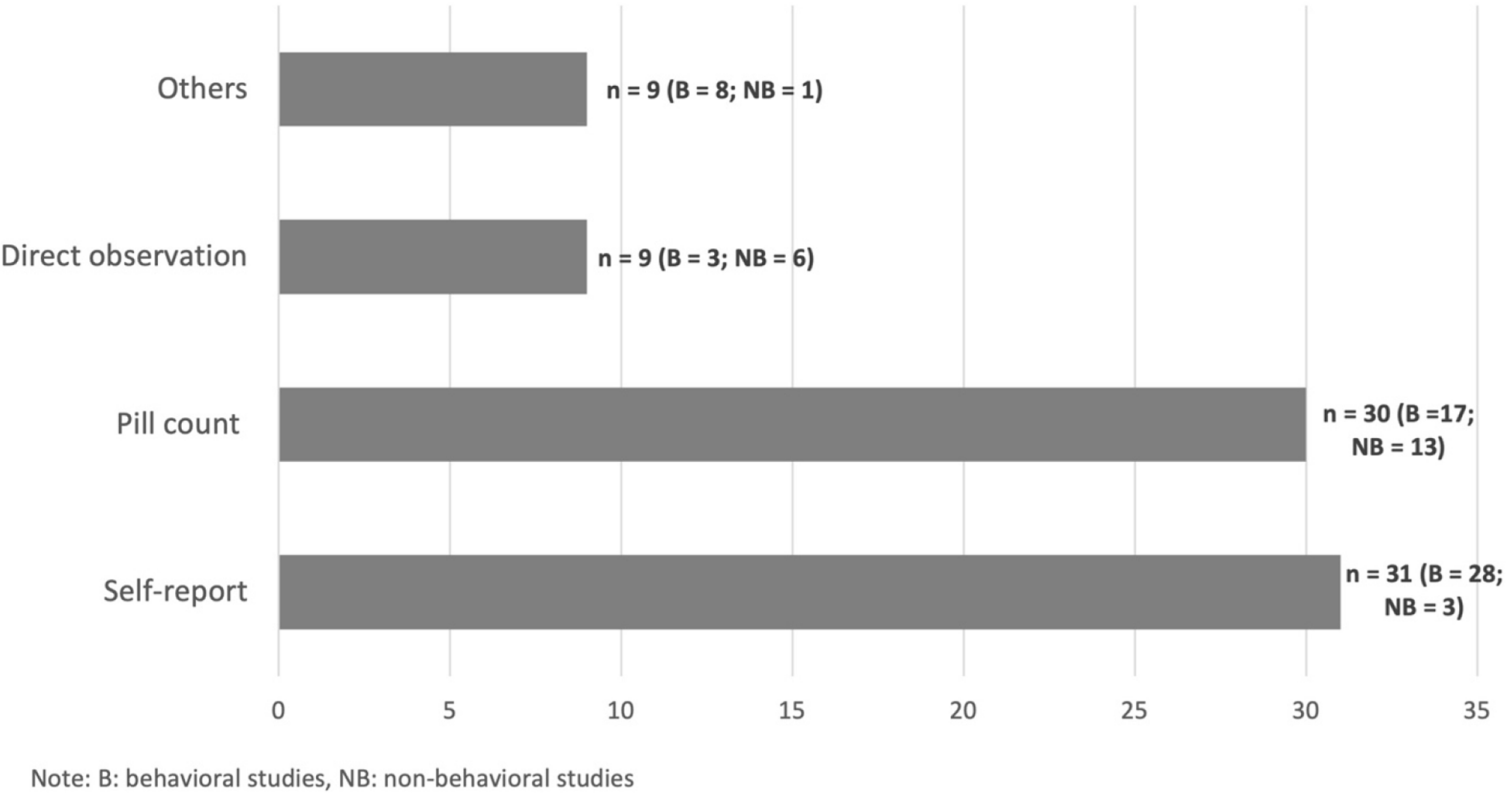
Frequency of adherence measurement methods among included studies

The analysis of measurement methods sheds light on the diverse approaches employed to assess adherence to micronutrient supplementation. Self-report was the most predominant approach, constituting 56%(*n* = 33) of overall studies. When comparing between study types, self-report was more common in behavioral studies(*n* = 33, 70%) than in non-behavioral studies(*n* = 3, 19%) (Fig. 2). A study that conducted training of community health volunteers in Nepal adapted the international household survey questionnaires, such as Demographic and Health Surveys, and asked the number of calcium tablets consumed during last pregnancy [62]. It is worth noting that most self-report measures took account of women’s pregnancy status and time period between supplement receipt and data collection. A study in Kenya recruited pregnant women in health facilities who confirmed receiving IFA tablets at ANC visit [68]. This study then conducted surveys after 4 to 6 weeks, asking about consumption in prior month with 4-point Likert scale responses. Those who responded ‘‘almost always’’ were classified as high adherers. Other studies, such as a community-based study in Kenya [43], had one-week time span in the measure to mitigate recall bias.

Pill count emerged as the next common method(n = 30,51%). Non-behavioral studies(n = 13, 81%) were largely relying on pill counts during health clinic or home visit, counting remining pills in the bottle. In many cases, data collection timing was designed to correspond to supplement provision. A study in Cambodia provided supplements to women at health facilities every month during which the bottle provided at the last visit was checked [20]. Often, pill count allowed data collectors to inquire about reasons for missed doses or directly observe supplement intake on the spot. In a calcium supplementation trial in Gambia, women were given color-coded tickets that were then brought to health facilities to exchange for daily tablets [25]. The study team counted tickets to measure adherence and asked about the barriers experienced by low-adherers.

In addition, direct observation was employed in 15% of the overall studies, which was more common in non-behavioral studies(*n* = 6, 38%) than behavioral studies(*n* = 3, 7%). Several studies employed direct observation not only as a data collection method but also as an intervention strategy. A behavioral study in India assigned an adult “observer” in the same community to observe and record pregnant women’s daily intake of IFA tablets [33]. Other identified methods of measurement included health clinic records and self-monitoring cards filled out by women.

While not common, medication event monitoring systems (MEMS) were used as an objective method of adherence assessment. MEMS automatically record the date and time a pill bottle is opened, providing a time-stamped record of use. Only two studies (3.4% of the total) employed this method, and both were behavioral interventions. Omotayo et al. [67] reported that MEMS revealed substantially lower adherence than self-reports, indicating that self-reported intake likely overestimated true adherence.

Additionally, many studies utilized multiple measurement methods, often termed data triangulation, to enhance the robustness of adherence assessments. A study in Vietnam used pill count along with direct observation by health workers [14]. Twenty-five(42%) of the included studies used more than one measure, but no studies used more than two. The purpose of using multiple methods was to validate and compare data across various sources, but limited description was available about if and how discrepancies were resolved.

Of the included studies, most measured adherence uniformly across participants regardless of micronutrient status. For example, women enrolled with iron deficiency anemia were all provided supplementation, and adherence was tracked using standard methods such as pill counts, self-reports, or direct observation. The only exception was Bah et al. [28], which employed a hepcidin-guided screen-and-treat approach. In this study, participants’ hepcidin levels were measured, and only women with low hepcidin were given iron supplements. Adherence was therefore assessed only in this subgroup using pill count, while women with high hepcidin were not prescribed iron and did not have adherence measured.

### Monitoring

We defined adherence monitoring as “regular tracking of supplement intake among participants that may be counted toward adherence measurement.” Monitoring tended to occur regularly during the intervention, beyond certain data collection time points(e.g., pre-post assessments), to encourage consistent supplement consumption. We also relied on studies’ own definition of monitoring to understand how monitoring activities were implemented. Most studies monitored supplementation as part of adherence measurement using the same measures. All non-behavioral studies regularly monitored consumption patterns mostly through pill count. Of the behavioral studies, 17 of them monitored adherence, employing a more diverse range of monitoring strategies. With self-report as the predominant method, some studies provided women with monitoring calendars to track their own supplementation progress.

### Studies with significant adherence outcomes

Out of 59 included studies, 31(53%) reported significant improvements in adherence outcomes. Of the studies with significant outcomes, 74% did not provide adherence definition or reporting cutoffs. Also, a majority of the studies with significant outcomes were conducted in lower-middle income countries(94%) rather than low-income countries(6%). Approximately 55% of the studies did not measure the impact of supplementation on health outcomes. Comparing adherence outcomes by study type further offered a nuanced understanding of data. Five(31%) non-behavioral studies showed significant adherence outcomes, whereas 26(60%) of behavioral studies showed significant adherence outcomes. These studies adopted various definitions of adherence and were mostly based on self-report(57%).

### Risk-of-bias assessment

Additional file 2 illustrates the evaluation of bias risk, as assessed by the authors, across a total of 59 studies comprising 23 RCT, 13 CRCT, and 23 non-randomized controlled trials (NRCT). In relation to the evaluation of adherence measurement, within the group of 23 RCT, 17 received a low risk of bias score, 1 raised some concerns, and 5 were attributed a high risk of bias score. Concerning the CRCT, 5 were appraised as having a low risk of bias, 2 exhibited some concerns, and 6 were classified as high risk of bias. As for the NRCT, 4 were designated as low risk of bias, 15 were deemed to carry a moderate risk of bias, 2 were identified as having a serious risk of bias, and 2 lacked available information for assessment.

## Discussion

Our systematic review examined studies that implemented micronutrient supplementation interventions in LMICs and empirically measured adherence to understand how adherence had been defined, measured, and assessed across the literature. Understanding how adherence is defined in a study has important implications for interpreting data on program effectiveness and making informed decisions.

We discovered that adherence has been commonly defined as taking $\ge$ 80% of recommended supplements, particularly among non-behavioral studies. This is consistent with the historically accepted threshold in the medication treatment literature based on Haynes’s early empirical definition of adherence used to dichotomize hypertensive patients who consumed sufficient medication and those who deviated from recommendations [73]. While this 80% threshold has been assumed to reflect both clinical efficacy and practicality in medicine, it is often adopted a priori by researchers without testing its relevance to micronutrient supplementation or examining whether alternative thresholds may be more appropriate for maternal nutrition interventions. As Gellad and colleagues emphasize, adherence is not a single construct but a process involving initiation, implementation, and discontinuation; yet, most studies reduce it to a single measure, such as the proportion of days covered or tablets consumed, often with an arbitrary cutoff of 80%. This reliance reflects more of a methodological convention than an empirically validated standard, since adherence is rarely defined on the basis of biological mechanisms or tailored to the intervention being studied [74]. A systematic review of studies that determined the relationship between adherence rates and clinical outcomes showed that only one out of six studies proved 80% as a valid threshold [75], underscoring the limited biological justification for applying this threshold to micronutrient supplementation programs [76–78].

While behavioral studies included in our review tended to adopt more varying definitions and cutoffs, ranging from 50 to 90% rates or ordinal categories, such as ‘high’, ‘medium’, and ‘low’, evidence behind these definitions was also scarce. The use of arbitrary cutoffs and dichotomization is prone to biases and substantial misinterpretation of data unless accompanied by a comprehensive presentation of data (e.g., presenting ordinal categories with a distribution plot) [75, 79, 80]. We recommend that researchers continue to use thresholds when appropriate, but ensure they are clearly justified, evidence-based, and presented alongside detailed data distributions to allow accurate interpretation and informed decision-making. Using evidence-based thresholds is imperative not only to avoid overestimation of program impacts but also to ensure accurate identification of individuals who would most benefit from an intervention to reach a desirable level of adherence and health. Evidence on thresholds validated for the population, micronutrients, and health outcomes of interest may properly guide the conceptualization and operationalization of adherence.

Consistent with prior evidence [6], our study also found that adherence measurement methods varied widely in the literature. Pill count and self-report were most common. Studies conducted in health facilities and non-behavioral studies largely relied on pill count, while most home-based and behavioral studies administered self-report measures. Previous studies argued that pill count is objective and sensitive to temporal changes, allowing for more accurate adherence measurement than self-report [81–83]. Indeed, evidence implied that self-report is suboptimal due to high misclassification and overestimation of adherence and low associations with clinical outcomes [84–87].

Social desirability and recall biases raise validity concerns when it comes to self-reports. However, it is often the most practical and feasible option in LMIC settings because other measures that are deemed “objective” raise challenges in their applicability. For instance, implementing pill counts imposes an onus on the women and the assessor and faces greater risks when pill bottles are misplaced [88]. How pills are counted and what measures are taken to maintain good hygiene during the count are also important considerations. Electronic monitoring, such as MEMS, is often impractical for large-scale projects due to its high cost and the fact that it tracks only container openings rather than actual supplement intake. Secondary data, such as pharmacy refills or health records, become unreliable due to weak health systems and infrastructure.

Thus, future research may merit employing strategies for enhancing self-report measures. A review article on the assessment of medication adherence in low-resource settings suggested strengthening interview skills among data collectors and developing instruments that reflect interviewees’ health literacy [88]. Additionally, Stirratt and colleagues provided recommendations for improving measurement validity in a LMIC-based program [87]: 1) using a 30-day recall period rather than shorter intervals while allowing room for adjusting the period depending on cognitive capacity among individuals, 2) adding a question that asks about global estimates of adherence to mitigate ceiling effects, as people tend to make an overall appraisal of their adherence rather than attentively counting doses taken or missed [89], 3) having external data collectors separate from study staff members who previously offered adherence services for participants, 4) including a statement in the survey that normalizes nonadherence to minimize social desirability bias, and 5) establishing high adherence thresholds or cutoffs to take account of overestimation of intake. We additionally encourage the use of data triangulation, as recommended by the WHO report [5]. The validity of selecting multiple methods is further optimized when they measure the same behavior over an identical time frame [74], of which most of the included studies fell short.

There is a need to make a distinction between adherence outcomes from non-behavioral studies and that of behavioral studies to better decipher the reported findings. The former was commonly conducted as clinical trials, aiming to maintain “sufficient” adherence across intervention and control groups to accurately assess treatment(e.g., types of micronutrients and regimens) effects on health, so some studies flagged group differences as limitations. Even so, measuring temporal changes in adherence and differences between study groups may provide critical insight into whether certain regimens, doses, and programmatic environments are acceptable to women and feasible in the longer term. Yet, we observed that 44% of the non-behavioral studies did not provide any empirical evidence beyond reporting average adherence rates while claiming the intervention was acceptable and ensured desirable adherence throughout the intervention. An article argued that clinicians and researchers often fail to recognize that adherence changes over time by choosing to average adherence data into overall rates even when it is measured repeatedly and longitudinally [74]. Incorporating advanced statistical methods, such as group‐based trajectory models, helps identify patterns of adherence among individuals who require tailored intervention strategies at different time points [90–92].

In contrast, behavioral studies applied more segmented analytical and reporting approaches, such as providing group differences at each visit or reporting within-group changes over time. Even though behavioral research seemed to acknowledge that adherence is not a static construct, most of the studies tended to measure adherence only once or twice, which partly led to having questions built on extreme time intervals(e.g., during entire pregnancy or prior week). Not surprisingly, routine data collection may not have been feasible for many programs since most were quasi-experimental studies conducted in less-controlled environments such as women’s homes. Nevertheless, we stress the need for routine data collection throughout an intervention to minimize potential biases (e.g., recall bias) and capture variations in adherence over time. A behavioral study in Kenya [67] did multiple home visits across six weeks to capture any variations in adherence, while another behavioral study in India conducted two rounds of surveys [31].

Mobilizing community resources, including community health workers and women’s social networks, establishing partnerships with health facilities and institutions, and using multiple sources for data collection may contribute to the robustness of adherence outcomes data. For example, some studies used women’s groups, particularly self-help groups, to provide maternal and newborn health messaging and to strengthen women’s social networks for reinforcing adherence and healthy practices [31, 32]. Also, other studies partnered with health facilities to deliver supplements and reinforce adherence, including interventions embedded within ANC services [23, 26, 29, 46]. A few other studies strengthened adherence measurement by using multiple data sources, such as combining self-reports with pill counts, electronic monitors, or biomarker assessments, thereby reducing bias from reliance on a single method [28, 60, 70].

Overall, most studies (69%) in our review were conducted for pregnant or recently delivered women, aligning with existing national health programs and policies for maternal supplementation in LMICs. Pregnancy represents a relatively short period during which individuals are often highly motivated to adhere to supplementation guidelines, which may not reflect adherence behaviors at other life stages [93]. As a result, nonpregnant women or adolescent girls could have been inadvertently overlooked in micronutrient supplementation research despite persisting malnutrition and high risks of morbidities later in life [94, 95]. This population may exhibit unique behavioral patterns due to social and cultural circumstances. Nonpregnant women may not have proper social support since supplements are perceived as unnecessary [96], and adolescent girls may be experiencing greater social stigma in the community, as supplement consumption is often mistakenly tied with early pregnancy or viewed as contraception. Shifting community norms about the supplementation needs of nonpregnant women and adolescents and exploring their challenges are urgently warranted in future research.

While not the primary focus of this review, among the included articles, we found that community norms and myths about supplements, environments in health facilities, financial instability among women, and distance to health facilities were cited as major barriers in low-income country-based studies without significant outcomes. We recommend conducting thorough formative assessments of facilitators and barriers to adherence among target groups to inform the development of an intervention. The RANI project in India developed a social norms-based intervention based on formative research data [97] on how unequal gender norms affected access to supplements among women of reproductive age, subsequently leading to improvement in IFA adherence in the intervention arm [31, 32]. The RANI project was the only study in our review that conducted a distinct formative research phase. In contrast, Omotayo et al. [67] employed the Trials of Improved Practices (TIPs) approach, in which the study itself functioned as formative research to assess the feasibility and acceptability of adherence partners, but this was not preceded by a separate formative phase. All other studies tested interventions without reporting formative research, highlighting a potential gap in tailoring adherence interventions to local contexts.

### Limitations

Our systematic review has several limitations. We excluded studies that measured adherence but did not report adherence outcomes. This could have rendered our findings on adherence measurement rather incomprehensive. Also, efficacy trials were largely eliminated given our aim to explore how adherence was handled in public health interventions. Including these studies would have confounded measurement of adherence(the behavior) with its outcomes(serum state). Third, our risk-of-bias assessment was performed based on information available. The results may be subject to errors if studies were conducted as protocol but simply did not report the procedure in entirety. Additionally, we acknowledge that the classification of non-behavioral and behavioral studies was not clear-cut, as several studies included elements of both. The decision was made through close reading of study objectives and discussion among four researchers to avert misclassification. Also, despite the increasing global efforts to promote MMS among women, most of the included projects were designed for IFA. This partly signifies a lack of rigorous methodology of adherence to MMS recommendations. We urge researchers to employ evidence-based approaches to defining, measuring, and assessing MMS adherence to meticulously ascertain program effectiveness. Furthermore, our analysis and reporting of adherence outcomes were based on studies’ reports of changes or improvements in adherence. However, many employed loose definitions and relied on a single measurement method, which may have led to overestimation, as partly highlighted in our risk-of-bias assessment results. Finally, we only included studies that were published in 2022. More recent work, which was not captured in this paper, may have introduced other means of measurement–an issue particularly relevant for MMS, which is being rolled out in many countries as we write this paper.

To our knowledge, this study is the first to review methodological approaches to micronutrient supplementation adherence among women of reproductive age in LMICs. By examining various aspects of measurement methodology—including adherence definitions, measurement methods, monitoring practices, and outcomes—across essential micronutrients for women, we provide critical recommendations for researchers and practitioners to enhance scientific efforts in supplementation programs.

## Conclusions

Understanding how maternal micronutrient supplementation programs have defined, measured, and assessed adherence is crucial for interpreting data comprehensively. While standardizing methodological approaches may be challenging due to unique programmatic factors and the multidimensional contexts that shape micronutrient supplementation behaviors among women across countries, this study provides practical recommendations to enhance scientific rigor in adherence data. Pill-count methods have often been used as a point of reference for assessing adherence, which does provide data without having to rely on self-reports (and associated biases), we note that this method is applicable only when medicinal counts are relevant, and it brings up other challenges (its intrusiveness, concerns about hygienically counting the pills, etc.). For this reason, and acknowledging that there is not a single “gold-standard,” we support the recommendation put forth by Burleson and colleagues [98] – to rely on triangulation rather than on a single assessment.

This review highlights potential programmatic gaps in low-income countries and a lack of focus on nonpregnant women and adolescents’ supplementation behaviors. Strengthening methodological approaches and broadening attention to all women of reproductive age in research can help public health practitioners, decision-makers, and donors make informed decisions to effectively address nutritional deficiencies among women in LMICs.

## Supplementary Information

Supplementary Material 1.

Supplementary Material 2.

## Data Availability

The datasets used and analyzed during the current study are available from the corresponding author on reasonable request.

## Abbreviations

ANC: Antenatal care
CRCT: Cluster randomized controlled trial
IFA: Iron and folic acid
LMICs: Low-and middle-income countries
MMS: Multiple micronutrient supplementation
NRCT: Non-randomized controlled trial
RCT: Randomized controlled trial
