# Supplementary material for: A systematic review of methodological approaches and measurement of adherence to micronutrient supplementation among women of reproductive age in low- and middle-income countries
Source: BMC Public Health. 2025 Dec 13;26:313. doi: 10.1186/s12889-025-24944-x (PMC12836925; doi:10.1186/s12889-025-24944-x)
Supplement: Supplementary file 1 — Supplementary Material 1. [file 12889_2025_24944_MOESM1_ESM.docx]

**Additional file 1. Search strategy on Pubmed**

Concept #1 – Women of reproductive age

"pregnant women"[mesh] OR "women"[mesh] OR "pregnancy"[mesh] OR "adolescent mothers"[mesh] OR "mothers"[mesh] OR "maternal health"[mesh] OR "pregnant wom*"[tw] OR "women"[tw] OR "woman"[tw] OR "reproductive age"[tw] OR "mother*"[tw] OR "maternal health"[tw] OR “maternal nutrition”[tw]

Concept #2 – Micronutrients

"iron"[mesh] OR "folic acid"[mesh] OR "calcium"[mesh] OR "micronutrients"[mesh] OR "dietary supplements"[mesh] OR "iron"[tw] OR "folic acid"[tw] OR "calcium"[tw] OR "micronutrient supplement*"[tw] OR "micronutrient*"[tw] OR "dietary supplement*"[tw] OR "multiple micronutrient supplement*"[tw] OR “ifa”[tw] OR “mms”[tw] OR “mmn”[tw]

Concept #3 – Supplementation behavior

"treatment adherence and compliance"[mesh] OR "medication adherence"[mesh] OR "medication compliance"[tw] OR "drug adherence"[tw] OR "drug compliance"[tw] OR “behavior modification”[tw] OR “behavior change”[tw] OR "adhere*"[tw] OR “compliance”[tw] OR “uptake”[tw] OR “coverage”[tw] OR “consumption”[tw] OR “use”[tw]

Concept #4 – LMIC

afghanistan[tw] OR algeria[tw] OR angola[tw] OR bangladesh[tw] OR benin[tw] OR bhutan[tw] OR bolivia[tw] OR burkina faso[tw] OR burundi[tw] OR cabo verde[tw] OR cambodia[tw] OR cameroon[tw] OR central african republic[tw] OR chad[tw] OR comoros[tw] OR congo[tw] OR cote d'ivoire[tw] OR djibouti[tw] OR egypt[tw] OR eritrea[tw] OR el salvador[tw] OR eswatini[tw] OR swaziland[tw] OR ethiopia[tw] OR gambia[tw] OR ghana[tw] OR guinea[tw] OR guinea bissau[tw] OR haiti[tw] OR honduras[tw] OR india[tw] OR indonesia[tw] OR iran[tw] OR kenya[tw] OR north korea[tw] OR democratic people's republic of korea[tw] OR kyrgyzstan[tw] OR kyrgyz republic[tw] OR laos[tw] OR lao pdr[tw] OR lao people's democratic republic[tw] OR lebanon[tw] OR lesotho[tw] OR liberia[tw] OR madagascar[tw] OR malawi[tw] OR mali[tw] OR micronesia[tw] OR kiribati[tw] OR mauritania[tw] OR mongolia[tw] OR morocco[tw] OR mozambique[tw] OR myanmar[tw] OR nepal[tw] OR nicaragua[tw] OR niger[tw] OR nigeria[tw] OR pakistan[tw] OR papua new guinea[tw] OR philippines[tw] OR rwanda[tw] OR samoa[tw] OR sao tome and principe[tw] OR senegal[tw] OR sierra leone[tw] OR solomon island*[tw] OR somalia[tw] OR south sudan[tw] OR sri lanka[tw] OR sudan[tw] OR syria[tw] OR syrian arab republic[tw] OR tajikistan[tw] OR tanzania[tw] OR timor leste[tw] OR east timor[tw] OR togo[tw] OR tunisia[tw] OR uganda[tw] OR ukraine[tw] OR uzbekistan[tw] OR vanuatu[tw] OR vietnam[tw] OR west bank[tw] OR gaza[tw] OR palestine[tw] OR yemen[tw] OR zambia[tw] OR zimbabwe[tw] OR global south[tw] OR africa south of the sahara[tw] OR sub saharan africa[tw] OR subsaharan africa[tw] OR central africa[tw] OR north africa[tw] OR northern africa[tw] OR magreb[tw] OR maghrib[tw] OR sahara[tw] OR southern africa[tw] OR east africa[tw] OR eastern africa[tw] OR west africa[tw] OR western africa[tw] OR west indies[tw] OR indian ocean islands[tw] OR caribbean[tw] OR central america[tw] OR latin america[tw] OR south america[tw] OR central asia[tw] OR north asia[tw] OR northern asia[tw] OR southeastern asia[tw] OR south eastern asia[tw] OR southeast asia[tw] OR south east asia[tw] OR western asia[tw] OR east europe[tw] OR eastern europe[tw] OR developing country[tw] OR developing countries[tw] OR developing nation[tw] OR developing nations[tw] OR developing population[tw] OR developing populations[tw] OR developing world[tw] OR less developed country[tw] OR less developed countries[tw] OR less developed nation[tw] OR less developed nations[tw] OR less developed world[tw] OR lesser developed countries[tw] OR lesser developed nations[tw] OR under developed country[tw] OR under developed countries[tw] OR under developed nations[tw] OR under developed world[tw] OR underdeveloped country[tw] OR underdeveloped countries[tw] OR underdeveloped nation[tw] OR underdeveloped nations[tw] OR underdeveloped population[tw] OR underdeveloped populations[tw] OR underdeveloped world[tw] OR middle income country[tw] OR middle income countries[tw] OR middle income nation[tw] OR middle income nations[tw] OR middle income population[tw] OR middle income populations[tw] OR low income country[tw] OR low income countries[tw] OR low income nation[tw] OR low income nations[tw] OR low income population[tw] OR low income populations[tw] OR lower income country[tw] OR lower income countries[tw] OR lower income nations[tw] OR lower income population[tw] OR lower income populations[tw] OR underserved countries[tw] OR underserved nations[tw] OR underserved population[tw] OR underserved populations[tw] OR under served population[tw] OR under served populations[tw] OR deprived countries[tw] OR deprived population[tw] OR deprived populations[tw] OR poor country[tw] OR poor countries[tw] OR poor nation[tw] OR poor nations[tw] OR poor population[tw] OR poor populations[tw] OR poor world[tw] OR poorer countries[tw] OR poorer nations[tw] OR poorer population[tw] OR poorer populations[tw] OR developing economy[tw] OR developing economies[tw] OR less developed economy[tw] OR less developed economies[tw] OR underdeveloped economies[tw] OR middle income economy[tw] OR middle income economies[tw] OR low income economy[tw] OR low income economies[tw] OR lower income economies[tw] OR low gdp[tw] OR low gnp[tw] OR low gross domestic[tw] OR low gross national[tw] OR lower gdp[tw] OR lower gross domestic[tw] OR lmic[tw] OR lmics[tw] OR third world[tw] OR lami country[tw] OR lami countries[tw] OR transitional country[tw] OR transitional countries[tw] OR emerging economies[tw] OR emerging nation[tw] OR emerging nations[tw] OR afghanistan[mh] OR algeria[mh] OR angola[mh] OR bangladesh[mh] OR benin[mh] OR bhutan[mh] OR bolivia[mh] OR burkina faso[mh] OR burundi[mh] OR cabo verde[mh] OR cambodia[mh] OR cameroon[mh] OR central african republic[mh] OR chad[mh] OR comoros[tw] OR congo[tw] OR cote d'ivoire[tw] OR djibouti[tw] OR egypt[tw] OR eritrea[mh] OR el salvador[mh] OR eswatini[mh] OR swaziland[mh] OR ethiopia[mh] OR gambia[mh] OR ghana[mh] OR guinea[mh] OR guinea bissau[mh] OR haiti[mh] OR honduras[mh] OR india[mh] OR indonesia[mh] OR iran[mh] OR kenya[mh] OR north korea[mh] OR democratic people's republic of korea[mh] OR kyrgyzstan[mh] OR kyrgyz republic[mh] OR laos[mh] OR lao pdr[mh] OR lao people's democratic republic[mh] OR lebanon[mh] OR lesotho[mh] OR liberia[mh] OR madagascar[mh] OR malawi[mh] OR mali[mh] OR micronesia[mh] OR kiribati[mh] OR mauritania[mh] OR mongolia[mh] OR morocco[mh] OR mozambique[mh] OR myanmar[mh] OR nepal[mh] OR nicaragua[mh] OR niger[mh] OR nigeria[mh] OR pakistan[mh] OR papua new guinea[mh] OR philippines[mh] OR rwanda[mh] OR samoa[mh] OR sao tome and principe[mh] OR senegal[mh] OR sierra leone[mh] OR solomon island*[mh] OR somalia[mh] OR south sudan[mh] OR sri lanka[mh] OR sudan[mh] OR syria[mh] OR syrian arab republic[mh] OR tajikistan[mh] OR tanzania[mh] OR timor leste[mh] OR east timor[mh] OR togo[mh] OR tunisia[mh] OR uganda[mh] OR ukraine[mh] OR uzbekistan[mh] OR vanuatu[mh] OR vietnam[mh] OR west bank[mh] OR gaza[mh] OR palestine[mh] OR yemen[mh] OR zambia[mh] OR zimbabwe[mh]
