# Supplementary material for: A systematic review of methodological approaches and measurement of adherence to micronutrient supplementation among women of reproductive age in low- and middle-income countries
Source: BMC Public Health. 2025 Dec 13;26:313. doi: 10.1186/s12889-025-24944-x (PMC12836925; doi:10.1186/s12889-025-24944-x)
Supplement: Supplementary file 2 — Supplementary Material 2. [file 12889_2025_24944_MOESM2_ESM.docx]

**Additional file 2**

**Additional file 2a. Risk of bias analysis results for randomized controlled trials (Cochrane RoB2)**

| **Author, year** | **Randomization process** | **Deviations from intended interventions** | **Missing adherence data** | **Measurement of adherence** | **Selection of the reported result** | **Overall bias** |
| --- | --- | --- | --- | --- | --- | --- |
| Ariyani, 2021 | Low | Some concerns | High | Some concerns | Some concerns | High |
| Adaji, 2019 | Low | Low | Low | Low | Low | Low |
| Ahamed, 2018 | Low | Some concerns | Low | Low | Low | Some concerns |
| Bah, 2019 | Low | Low | Low | Low | Low | Low |
| Baxter, 2014 | High | Low | Low | High | Low | High |
| Bilimale, 2010 | Low | Some concerns | Low | Low | Some concerns | Some concerns |
| Byamugisha, 2022 | Low | High | High | High | Low | High |
| Compaoré, 2018 | Low | Some concerns | Low | Low | Some concerns | Some concerns |
| Duggan, 2014 | Low | Low | Low | Low | Low | Low |
| Goldberg, 2013 | Low | Low | Low | Low | Low | Low |
| Gonzalez-Casanova, 2017 | Low | Low | Low | Low | Low | Low |
| Gunaratna, 2015 | Low | Low | Low | Low | High | High |
| Heryadi, 2017 | Low | Some concerns | Low | Low | Low | Some concerns |
| Hofmeyr, 2019 | Low | Low | Low | Low | Low | Low |
| James, 2019 | High | High | Low | High | High | High |
| Karakochuk, 2017 | Low | Low | Low | Low | Low | Low |
| Khorshid, 2014 | Some concerns | Some concerns | Low | Low | Low | High |
| Nguyen, 2016 | Low | Low | Low | High | Some concerns | High |
| Nwaru, 2015 | High | Low | Low | High | Low | High |
| Ramakrishnan, 2016 | Low | Low | Low | Low | High | High |
| Srivastava, 2019 | Low | High | Low | Low | High | High |
| Taneja, 2021 | High | Low | Low | Low | Low | High |
| Vanobberghen, 2021 | Low | Low | Low | Low | Low | Low |

**Additional file 2b. Risk of bias analysis results for cluster randomized controlled trials (Cochrane RoB2 for cluster randomized controlled trials)**

| **Author, year** | **Randomization process** | **Timing of recruitment** | **Deviations from intended interventions** | **Missing adherence data** | **Measurement of adherence** | **Selection of the reported result** | **Overall bias** |
| --- | --- | --- | --- | --- | --- | --- | --- |
| Bharti, 2015 | Low | Low | Low | Low | High | Low | High |
| Ganjoo, 2022 | Low | Low | Low | Low | High | Low | High |
| Hanieh, 2013 | Low | Low | Some concerns | Low | Low | High | High |
| Harding, 2017 | Some concerns | Low | Low | Low | High | Some concerns | High |
| Martin, 2017 | Some concerns | Some concerns | High | Low | Some concerns | Low | High |
| Nguyen, 2021 | Low | Some concerns | Low | Low | Low | Low | Some concerns |
| Nguyen, 2017 | High | Low | Some concerns | Low | High | Low | High |
| Nguyen, 2018a | Low | Low | Some concerns | Low | Low | Some concerns | High |
| Nguyen, 2018b | Some concerns | Low | Some concerns | Low | Some concerns | Low | High |
| Omotayo, 2018a | Low | Some concerns | Some concerns | Low | Low | Some concerns | High |
| Omotayo, 2017 | Low | Low | Low | High | High | Low | High |
| Sedlander, 2022 | Low | Low | Low | Low | High | Some concerns | High |
| Todd, 2019 | Low | Low | Low | Low | Low | Low | Low |

**Additional file 2c. Risk of bias analysis results for non-randomized studies (ROBINS-I)**

| **Author, year** | **Bias due to confounding** | **Bias in participant selection** | **Bias in classification of interventions** | **Deviations from intended interventions** | **Missing adherence data** | **Measurement of adherence** | **Selection of the reported result** | **Overall bias** |
| --- | --- | --- | --- | --- | --- | --- | --- | --- |
| Abioye, 2016 | Low | Low | Serious | Low | Low | Serious | Low | Serious |
| Anitasari, 2017 | Low | Moderate | Moderate | Low | Moderate | Moderate | Low | Moderate |
| Brasington, 2016 | Low | Low | Low | Low | Low | Moderate | Low | Moderate |
| Clermont, 2018 | Low | Low | Low | Low | No Information | Low | No Information | Low |
| Gamboa, 2020 | Serious | No Information | Moderate | Low | Serious | Moderate | Moderate | Serious |
| Hazra, 2020 | Moderate | Low | Moderate | Low | Low | Moderate | Low | Moderate |
| Kamau, 2020 | Low | Low | Moderate | Moderate | Moderate | Moderate | Serious | Serious |
| Klemm, 2020 | No Information | Low | Moderate | Low | Low | Serious | Moderate | Serious |
| Kung’u, 2018 | Moderate | Low | Low | Low | Moderate | Low | Low | Moderate |
| Kurzawa, 2021 | Moderate | Low | Moderate | Low | Low | Moderate | Low | Moderate |
| Morteza, 2017 | Moderate | Low | Low | Low | Low | Low | Moderate | Moderate |
| Nahrisah, 2020 | Low | Low | Moderate | Low | Low | Moderate | Low | Moderate |
| Noronha, 2013 | Moderate | Moderate | Moderate | Low | Low | Moderate | Low | Moderate |
| Omotayo, 2018b | Critical | Low | Low | Moderate | No Information | Low | Serious | Critical |
| Ouedraogo, 2019 | Low | Low | Low | Low | Moderate | Moderate | Low | Moderate |
| Riang’a, 2020 | Serious | Moderate | No Information | No Information | No Information | No Information | Serious | Serious |
| Rukmaini, 2018 | Moderate | Low | Low | Low | Low | Moderate | Low | Moderate |
| Sharma, 2016 | Serious | Low | Low | Low | Low | Moderate | Low | Serious |
| Shivalli, 2015 | Low | Low | Low | Low | Low | Moderate | Moderate | Moderate |
| Surtimanah, 2019 | Low | Moderate | Moderate | Low | Low | Moderate | Low | Moderate |
| Thapa, 2016 | Low | Low | Serious | No Information | Low | No Information | Low | Serious |
| Walia, 2020 | Low | Low | Moderate | Low | Low | Moderate | Low | Moderate |
| Wulandari, 2022 | Moderate | Low | Moderate | Low | Low | Moderate | Low | Moderate |
